# A general modeling and visualization tool for comparing different members of a group: application to studying tau-mediated regulation of microtubule dynamics

**DOI:** 10.1186/1471-2105-9-339

**Published:** 2008-08-12

**Authors:** Arnab Bhattacharya, Sasha Levy, Adria LeBoeuf, Michelle Gaylord, Leslie Wilson, Ambuj K Singh, Stuart C Feinstein

**Affiliations:** 1 Department of Computer Science and Engineering, Indian Institute of Technology (I.I.T.), Kanpur, India; 2 Department of Computer Science, University of California at Santa Barbara, Santa Barbara, CA, USA; 3 Center for Comparative Functional Genomics, New York University, New York, NY, USA; 4 Howard Hughes Medical Institute and Laboratory of Sensory Neuroscience, Rockefeller University, New York, NY, USA; 5 Department of Molecular, Cellular and Developmental Biology, University of California at Santa Barbara, Santa Barbara, CA, USA

## Abstract

**Background:**

Innumerable biological investigations require comparing collections of molecules, cells or organisms to one another with respect to one or more of their properties. Almost all of these comparisons are performed manually, which can be susceptible to inadvertent bias as well as miss subtle effects. The development and application of computer-assisted analytical and interpretive tools could help address these issues and thereby dramatically improve these investigations.

**Results:**

We have developed novel computer-assisted analytical and interpretive tools and applied them to recent studies examining the ability of 3-repeat and 4-repeat tau to regulate the dynamic behavior of microtubules in vitro. More specifically, we have developed an automated and objective method to define growth, shortening and attenuation events from real time videos of dynamic microtubules, and demonstrated its validity by comparing it to manually assessed data. Additionally, we have used the same data to develop a general strategy of building different models of interest, computing appropriate dissimilarity functions to compare them, and embedding them on a two-dimensional plot for visualization and easy comparison. Application of these methods to assess microtubule growth rates and growth rate distributions established the validity of the embedding procedure and revealed non-linearity in the relationship between the tau:tubulin molar ratio and growth rate distribution.

**Conclusion:**

This work addresses the need of the biological community for rigorously quantitative and generally applicable computational tools for comparative studies. The two-dimensional embedding method retains the inherent structure of the data, and yet markedly simplifies comparison between models and parameters of different samples. Most notably, even in cases where numerous parameters exist by which to compare the different samples, our embedding procedure provides a generally applicable computational strategy to detect subtle relationships between different molecules or conditions that might otherwise escape manual analyses.

## Background

### Statement of Problem

Innumerable biological investigations require comparing different members of a collection of entities with respect to one or more properties. The conclusions to be drawn from such studies are based on an analysis of the degree of similarity or dissimilarity among the different members. For example, one might compare the activity of different isoforms or fragments of a protein of interest, or compare wild type protein(s) with various mutant versions of a protein that causes a disease state. Many additional examples come from comparisons of data sets derived from microarray and proteomics studies, as well as population genetics. Given the technical advances of recombinant DNA technology and the explosion in genomics over the past few years, it is a certainty that the number of these sorts of comparative studies, and the number of entities to be compared within each study, will increase dramatically in the near future. Unfortunately, the vast majority of such comparative studies are currently performed manually, with investigators searching for similarities and dissimilarities among different test entities "by eye". This is especially difficult when each member of the collection is being characterized by multiple criteria. The analytical process is time consuming, likely to miss subtleties and is susceptible to inadvertent bias and human errors. Development and application of computer-assisted modeling and visualization can provide extraordinarily valuable data analyses and interpretive tools for assessing relationships among different members in a study.

### Microtubules and Microtubule Dynamics

Microtubules represent one of the three main components of the eukaryotic cellular cytoskeleton [1]. They are hollow, unbranched cylinders, formed by the non-covalent association of *αβ tubulin *dimer subunits. Microtubules serve a wide variety of essential structural and transport functions, including the segregation of chromosomes during cell division and the transport of vesicular cargo up and down long axonal processes in neurons.

Microtubules are highly dynamic structures, gaining and losing tubulin dimer subunits by a stochastic process known as *dynamic instability *[2,3]. A large body of data, both pharmacological and somatic cell genetics, has led to the conclusion that proper regulation of microtubule dynamics is essential in order for microtubules to perform their many critical cellular functions (for review, see [4]). For example, the effectiveness of the anti-cancer drug taxol derives from its ability to suppress microtubule dynamics, thereby interfering with the ability of cancer cells to proliferate [5]. Given the importance of properly regulated microtubule dynamics, it is not surprising that cells have evolved a host of regulatory proteins that finely tune microtubule dynamics, including tau, MAP2, MAP4, SCG10 and stathmin.

### The Microtubule Associated Protein Tau

**Figure 1:**
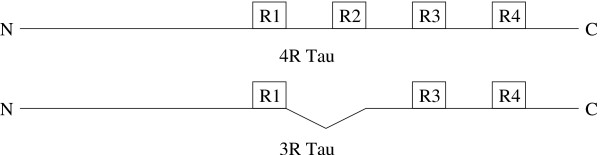
**Isoforms of tau**. Schematic of two classes of tau isoforms – 4-repeat tau and 3-repeat tau. In 3-repeat tau, the region between repeats 1 and 2 and the second repeat structures are missing by virtue of alternative RNA splicing.

The microtubule associated protein *tau *is essential for the normal development and maintenance of the nervous system [6-8]. It binds directly to microtubules [9,10], and its ability to regulate microtubule dynamics [11-13] is itself tightly regulated by both alternative RNA splicing [14] and phosphorylation (for review, see [15]). Alternative RNA splicing leads to the synthesis of two classes of tau, known as 3-repeat tau and 4-repeat tau (See Figure 1 for a schematic). Whereas normal human fetal brain expresses only 3-repeat tau, adult human brain expresses approximately equal amounts of 3-repeat and 4-repeat tau. Despite this dramatic developmental shift in expression profiles, the functional and mechanistic differences between 3-repeat and 4-repeat tau remain poorly understood. While it is well-established that 4-repeat tau is a more potent regulator of microtubule dynamics than 3-repeat tau, there have been indications over the years that the two classes of tau isoforms may also have inherent qualitative differences as well [12,16-19].

Abnormal tau action has long been correlated with neurodegeneration. Indeed, the classic neurofibrillary tangle pathology of Alzheimers and many related dementias are composed primarily of aberrant tau (for example, see [20]). In 1998, a direct cause and effect relationship between errors in tau action and/or regulation and neurodegeneration was established by the genetic linkage between mutations in the tau gene and FTDP-17, a fronto-temporal dementia with many similarities to Alzheimers disease [21-23]. Two classes of tau mutations have been described. The first collection of mutations are structural in nature, caused by various amino acid substitutions in tau. The second class of mutations are especially subtle and provocative – they are caused by errors in alternative tau RNA splicing that alter the expression ratio of otherwise normal 4-repeat and 3-repeat tau molecules. Specifically, rather than a ~50/50 ratio in adult human brain, the mutant ratio is closer to ~75/25. In both the structural and regulatory mutations, the result is early onset of neuronal cell death and dementia.

Unfortunately, the molecular mechanisms underlying tau-mediated neuronal cell death remain unclear. One widely held model suggests that errors in tau action lead to the aggregation of tau into neurofibrillary tangles, which are in turn cytotoxic [24]. An alternative model suggests that tau-mediated neuronal cell death results from the inability of tau to properly maintain microtubule dynamics within a narrow range of activities required for cell viability [4,13,19,25]. Additional models have also been proposed (see ).

### Computational Perspectives

**Figure 2:**
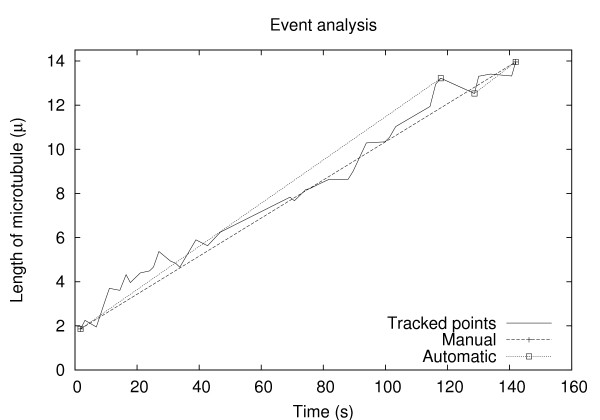
**Example life history plot**. An example of a typical "life history" plot of a microtubule, i.e., microtubule length as a function of time plot. The microtubule shown here is from the 3-repeat tau sample at a tau:tubulin molar ratio of 1:38.

To quantitatively investigate the regulation of microtubule dynamics under varying conditions (for example, with different tau isoforms or tau:tubulin molar ratios), cell biologists employ video microscopy to visualize and record images of dynamic microtubules in real time. For each condition being assessed, many different individual microtubules must be imaged, tracked and analyzed [19]. From the resulting microtubule "life history plots" (Figure 2), the dynamic behaviors of similarly treated microtubules can be determined, such as average growth or shortening rates. Subsequently, the behavior of microtubules under different conditions can be compared.

Computer-assisted methods are especially attractive for time series investigations of this sort. In the specific case of analyzing the regulation of microtubule dynamics, inadvertent bias and non-reproducibility in data interpretation among different labs and different investigators when defining the beginning and end points of individual growth, shortening or attenuation events could become significant. Despite the fact that these events are explicitly defined, investigators must make many judgment calls. In contrast, computer-assisted methods offer a faster and more objective assessment of the data. More importantly, these methods can also provide analytical tools as much as determine the fit of the data to various statistical models, thereby testing various conceptual representations of the underlying molecular mechanisms of action of the system under study. Modeling can also generate testable mechanistic predictions for subsequent investigations. In a general sense, sophisticated computational tools have the potential to make major contributions to many areas of biological research.

## Results

The main goal of this work is to develop general computational tools to quantitatively assess the differences among samples of interest and to visualize those differences in a manner that facilitates their comparison. The data being analyzed is derived from an earlier work in which video microscopy was used to visualize and assess the abilities of 3-repeat and 4-repeat tau to regulate various parameters of microtubule dynamics in *in vitro *reactions (Levy et al., 2005) [19]. Samples contained purified tubulin dimers and purified recombinant human tau. The two primary variables were (i) the presence of 3-repeat tau, 4-repeat tau or no tau, and (ii) the molar ratio of tau to tubulin. In vivo, the molar ratio of tau:tubulin varies from cell to cell. Further, the ratio can vary even among different regions within single cells, such as cell body versus axon versus growth cone. The different ratios examined are likely to span the range of biologically meaningful values [26].

### Automated Life History Plot Analysis

We first developed an automated method to identify the different events – growth, shortening, and attenuation ("pause") – on microtubule life-history plots, using a set of pre-defined rules (see the Methods section for details). We then compared the ability of this automated method to determine average microtubule growth rates with standard manual analysis, using data sets assessing the ability of tau to regulate microtubule dynamics from [19]. In this earlier work, microtubule dynamics were assayed under nine different experimental conditions. As seen in Table 1, the deviation between automatic and manually determined rates ranges from 3.33% to 12.68%, with an average deviation of 6.59%. Statistically, the differences between the manually determined values and the automatically measured values are not significant (as shown by the p-values), except for one condition. The p-value is computed by performing a t-test of the manually determined growth rates against the automatically computed ones for each condition (details are in the Methods section). More importantly, the relative order of the conditions do not change, and the degrees of separation are well maintained. Table 2 shows the comparison for the same set of conditions using a different tubulin preparation from [19]. Again, our automated method accurately recapitulates manual analysis with increased objectivity. Additionally, it markedly reduces the time required to conduct these investigations.

**Table 1:** Growth rates of tau conditions (sample 1)

Condition	Manual	Automatic	Difference	Deviation	P-value
3R-1:20	3.99	4.19	0.20	4.93%	0.15
3R-1:38	3.58	3.76	0.18	4.80%	0.05
3R-1:45	2.02	2.27	0.25	12.58%	0.05
3R-1:55	2.02	2.28	0.26	12.68%	0.03
4R-1:20	4.71	4.56	0.15	3.33%	0.85
4R-1:38	3.96	4.19	0.23	5.86%	0.15
4R-1:45	3.51	3.65	0.14	4.14%	0.23
4R-1:55	2.59	2.84	0.25	9.45%	0.05
No-Tau	2.30	2.53	0.23	10.13%	0.27

Average			0.21	6.59%	

The manual data is reproduced from Table III of (Levy et al., 2005) [19]. The third column shows growth rates (in *μm*/*min*) automatically computed from the manually tracked tips of the microtubules using an objective set of rules with no human interference. The relative ranks of the conditions remain the same.

**Table 2:** Growth rates of tau conditions (sample 2)

Condition	Manual	Automatic	Difference	Deviation	P-value
3R-1:20	3.90	4.02	0.12	3.07%	0.22
3R-1:38	2.67	2.87	0.20	7.49%	0.73
3R-1:45	2.16	2.39	0.23	10.65%	0.13
3R-1:55	2.34	2.47	0.13	5.56%	0.35
4R-1:20	4.93	4.99	0.06	1.22%	0.63
4R-1:38	4.39	4.63	0.24	5.47%	0.43
4R-1:45	3.87	3.87	0.00	0.00%	0.75
4R-1:55	3.25	3.47	0.22	6.77%	0.14
No-Tau	2.77	2.95	0.18	6.50%	0.36

Average			0.15	4.55%	

The manual data is reproduced from Table II of (Levy et al., 2005) [19]. The third column shows growth rates (in *μm*/*min*) automatically computed from the manually tracked tips of the microtubules using an objective set of rules with no human interference. The relative ranks of the conditions remain the same.

It is also important to note that inherent biological variability exists in the microtubule growth rate data. This likely results from biochemical variations between different tubulin preparations, such as different tubulin isoform expression ratios and/or varying degrees of post-translational modifications, such as phosphorylation, acetylation or tyrosination. For example, assuming each growth rate determination is within a variation of ± 0.1 *μm*/*min*, the rank order of the conditions for each of the two data sets is quite similar, although the 4R-1:55 and 3R-1:38 conditions are reversed (see Table 3). This inherent biological variability could limit the utility of some sophisticated and highly sensitive statistical models to make testable predictions regarding mechanisms underlying the regulation of microtubule dynamics. At the minimum, multiple data sets might be necessary and all predictions would need to be considered tentative until tested directly by other biological means.

**Table 3:** Rank order of different conditions

Rank	Condition (growth rate)	
	
	Table III of (Levy et al., 2005) [19] (Table 1 of this paper)	Table II of (Levy et al., 2005) [19] (Table 2 of this paper)
1	4R-1:20 (4.7)	4R-1:20 (4.9)
2	4R-1:38 (4.0)	4R-1:38 (4.4)
3	3R-1:20 (4.0)	3R-1:20 (3.9)
4	4R-1:45 (3.5)	4R-1:45 (3.9)
5	3R-1:38 (3.6)	4R-1:55 (3.3)
6	4R-1:55 (2.6)	3R-1:38 (2.7)
7	No-Tau (2.3)	No-Tau (2.8)
8	3R-1:45 (2.0)	3R-1:45 (2.2)
9	3R-1:55 (2.0)	3R-1:55 (2.3)

The ranks of the conditions remain almost the same across the two samples (considering a variation of 0.1 *μm*/*min*) except that the 4R-1:55 and the 3R-1:38 conditions are reversed.

### Modeling and Embedding Strategy

Next, we sought to develop mathematical and statistical models to capture different dynamic aspects of microtubule behavior and to embed them in a two-dimensional space for visualization and easy comparison of different conditions. We used the Sammon projection method [27] for embedding and visualization. In short, the embedding process displays each experimental condition with an (*x*, *y*) position; the relative distance between the (*x*, *y*) positions of any pair of experimental conditions corresponds to their relative degree of relatedness (details are in the Methods section). The conditions of interest can be compared based on numerous parameters and the computational method is applicable to all kinds of numerical parameters.

The outline of our method is as follows. First, the experimental measurements are analyzed based on an appropriate mathematical model. Then, an appropriate dissimilarity function is applied to calculate the relative distances between the models of each pair of conditions. Finally, the conditions are embedded on a two-dimensional space such that the inherent structure of the data is approximately preserved. This is achieved by assigning points (*x *and *y *coordinates) to the models such that the Euclidean distance between any pair of points in this space is as close to the original dissimilarity measure between their models as possible. Unlike principal component analysis (PCA) [28], this method works with any distance matrix. The quality of the embedding is measured by *distortion*. For ideal embeddings, where all dissimilarity values are maintained exactly as Euclidean distances in the embedded space, the distortion is 1. The details of the models, the dissimilarity functions, the embedding algorithm, and the distortion computations are presented in the Methods section.

### Two-Dimensional Embedding Analysis

#### Microtubule Growth Rate

**Figure 3:**
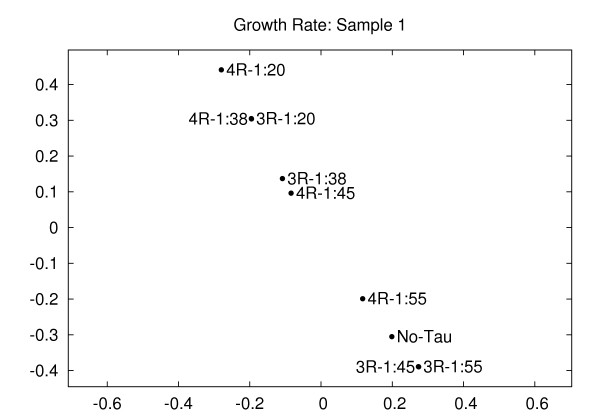
**Growth rate (sample 1)**. Embedding of the growth rates of tau conditions for sample 1 (corresponding to Table 1). The distortion is 1, indicating no error in embedding. The automatically computed growth rates maintain the relationship of the conditions as described in the Results section and in [19]. Distortion = 1.00.

As a proof-of-principle exercise, we used the automatically measured values from Table 1 and applied our embedding strategy to compare the abilities of each tau isoform to regulate the growth rate of microtubules. Since these growth rate data are one-dimensional, the distortion is 1, and the embedding procedure should yield a straight line. Figure 3 shows the two-dimensional embedding of the conditions. The requirement for the distances are fulfilled and the points are on a straight line. Additionally, consistent with [19], we observe that very low ratios of 3-repeat and 4-repeat tau:tubulin have opposite effects on the dynamic behavior of microtubules. More specifically, while 1:55 and 1:45 3-repeat tau and 1:55 4-repeat tau are all relatively close to the control (no-tau) point, the two 3-repeat tau conditions decrease the microtubule growth rate while the 1:55 4-repeat tau condition increases it as compared to the no-tau condition. Additionally, any increase in the tau:tubulin ratio beyond these low levels causes a relatively large increase in growth rate, since the distance between the no-tau point and all other tau points is relatively large. Thus, there are two clusters of growth rates rather than a simple linear relationship, consistent with a threshold effect. Further, as the tau:tubulin ratio increases (for both tau isoform classes), the difference with the no-tau point increases. Finally, for any given tau:tubulin ratio, 4-repeat tau is always more distant from the no-tau point than 3-repeat tau is; this demonstrates that 4-repeat tau is a more potent regulator of microtubule dynamics than 3-repeat tau. Thus, these data establish the validity of our automated life history analytical method and the two-dimensional embedding method.

**Figure 4:**
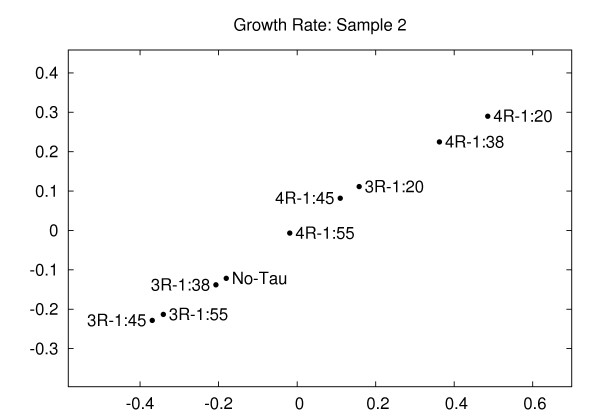
**Growth rate (sample 2)**. Embedding of the growth rates of tau conditions for sample 2 (corresponding to Table 2). The distortion is 1, indicating no error in embedding. The automatically computed growth rates maintain the relationship of the conditions as described in the Results section and in [19]. Distortion = 1.00.

Figure 4 shows the plot for another set of samples corresponding to the values in Table 2. This second sample corresponds to tubulin preparation 1 mentioned in Table II of [19]. The low ratios of 3-repeat and 4-repeat tau:tubulin behave similar to the control (no-tau) point. The higher ratios cluster separately.

#### Microtubule Growth Rate Distribution Histogram

Next, we used two-dimensional embedding to compare the effects of 3-repeat tau and 4-repeat tau upon the distribution of growth rates within the growing population of microtubules. As demonstrated in [19], a histogram analysis of control populations of growing microtubules yields two pools – a more abundant and slower growing pool and a less abundant and faster growing pool. Based on fitting mixture of two Gaussians to the histograms, the authors concluded that both tau isoforms cause an increase in the abundance of the faster growing pool and a decrease in the abundance of the slower growing pool, with 4-repeat causing the population change at lower tau:tubulin ratios than 3-repeat tau. Other than these conclusions, there was no other comparison possible between the histograms.

**Figure 5:**
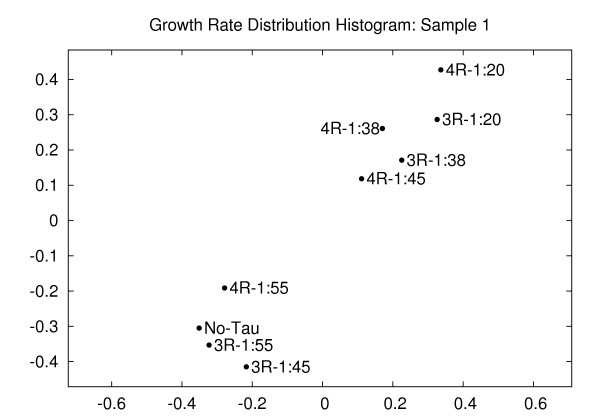
**Growth rate distribution histogram (sample 1)**. Embedding of the microtubule growth rate distributions at varying tau:tubulin molar ratios for sample 1. The growth behavior of microtubules for low molar ratios of tau:tubulin for both 4-repeat and 3-repeat taus are similar to those in no-tau conditions. In higher molar ratios, however, the behavior is quite different. Distortion = 1.84.

We subjected the growth rate distribution data to our two-dimensional embedding analysis method (Figure 5). Each distribution histogram had 19 bins (similar to the analysis in [19]), and the dissimilarities among the histograms were computed by the match distance [29]. Conceptually, the match distance takes into account both the height of a histogram bin and the spatial position of the bin in the histogram; two histograms that differ in far-off bins are more distant than histograms that differ in adjacent bins. The details of the procedure are presented in the Methods section. As was true for the growth rates (Figure 3), the histogram distribution data reveals that there are only minor differences among the control (no-tau) sample and low levels of tau (3-repeat tau at both 1:55 and 1:45 tau:tubulin ratio and 4-repeat tau at a 1:55 tau:tubulin ratio). Moreover, in a manner parallel to the growth rates, 3-repeat and 4-repeat tau regulate microtubule dynamics in different directions, as indicated by the fact that the 4-repeat tau (1:55 ratio) is closer to the no-tau point than it is to either of the 3-repeat tau (1:55 or 1:45) samples.

Additionally, it is also clear that 4-repeat tau is more potent than 3-repeat tau at any given tau:tubulin ratio (i.e., the distance between the 4-repeat tau point and the no-tau point is greater than the distance between the 3-repeat tau point and the no-tau point for all molar ratios). Finally, similar to the growth rate analysis in Figure 3, there are two clusters of behaviors rather than a continuum. One cluster contains the no-tau point and the lower tau:tubulin ratio samples and the other cluster contains the higher tau:tubulin ratio samples. Such non-linearity coupled with different functional effects could have significant mechanistic effects in the alternative RNA splicing class of tau FTDP-17 mutations in which relatively subtle increases in the 4-repeat tau concentration have dramatic consequences. By assessing the histogram landscape of the conditions, the two-dimensional embedding procedure complements the previous analyses using Gaussian mixture models [19]. The two-dimensional embedding plot is more sensitive in picking out the differences between a pair of conditions or among multiple conditions; on the other hand, it shows distances that lack physical meaning.

**Figure 6:**
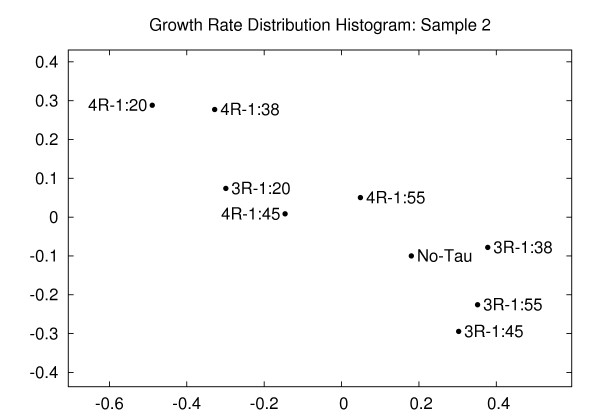
**Growth rate distribution histogram (sample 2)**. Embedding of the microtubule growth rate distributions at varying tau:tubulin molar ratios for sample 2. The growth behavior of microtubules for low molar ratios of tau:tubulin for both 4-repeat and 3-repeat taus are similar to those in no-tau conditions. In higher molar ratios, however, the behavior is quite different. Distortion = 1.69.

Figure 6 shows the corresponding embedding plot of the growth rate distribution histograms for another set of samples. This second sample corresponds to tubulin preparation 1 mentioned in Table II of [19]. Similar to the case presented in Figure 5, the low ratios of 3-repeat and 4-repeat tau:tubulin cluster together with the control (no-tau) point. The higher ratios of tau:tubulin induce shifts in the growth rates.

Additional file 1 shows the effect of the number of bins on the embedding plots. Histograms were generated by varying the number of bins from 4 to 29 in variations of 5. The plots show only minor differences. In all of them, the lower tau:tubulin ratios (4-repeat tau at 1:55, and 3-repeat tau at 1:55 and 1:45) and the control (no-tau) point are far away from the higher tau:tubulin ratios.

#### Microtubule Dynamics and Haar Wavelets

Finally, we compared the two-dimensional embeddings of the Haar wavelet features [30] to 3-repeat tau, 4-repeat tau and the control (no-tau) samples. Wavelets [31] are powerful statistical tools that are used for a wide range of applications, including signal description and data compression. One of the main advantages of wavelets is that they offer a simultaneous localization in both time and frequency domains. Further, they can provide a multi-resolution view of the original time-series by changing the width of the "window" over which the coefficients are computed. Haar wavelets [30] are the simplest and the fastest to calculate among all the different types of wavelet functions. The specific window sizes and the details of how the dissimilarities among the conditions are computed are described in the Methods section. Additional file 2 shows the plots for the two different samples. The disparity in the two plots likely arises from the inherent variability in the biological data. The first plot (corresponding to the data set presented in Table 1) suggests two distinct clusters, one corresponding to the 3-repeat tau conditions and the other to the 4-repeat tau conditions, consistent with the notion that 3-repeat and 4-repeat tau might interact with microtubules in qualitatively distinct manners. The lack of similar behavior for the second data set (see Table 2) makes the conclusions from the plots tentative, requiring independent corroboration.

We also used two-dimensional embedding to compare the effects of 3-repeat and 4-repeat tau with respect to the Markov Chain models. A Markov Chain (MC) [32] captures the underlying dynamics of the physical phenomena or entity by a generative model that emits a sequence of symbols. The primary advantage of Markov Chains over other models of time-series data is their ability to characterize an entire family of sequences. MCs are fairly easy to build, require a small set of sequences and allow very fast searching and comparison. There was no obvious clustering of points with respect to either the tau:tubulin ratio or 3-repeat tau versus 4-repeat tau (plot not shown). We used other time-series models as well, like the Lomb-Scargle periodograms [33,34] that can assess periodic behaviors (akin to Fourier analysis [35]) even in the presence of missing data and unequal sampling frequencies. Unfortunately, the embedding plot did not reveal any clear patterns, with the exception that the control (no-tau) point was on a distant corner of the plot and the tau samples with lower molar ratios of tau:tubulin are closer to the no-tau point than the samples with higher ratios (data not shown). Another class of models – the auto-regressive moving average (ARMA) models [36] – has often been used in analyzing time-series data. These models assume that the data is stationary, i.e., both the mean and the variance is fixed. Since the microtubules are clearly growing, we did not consider these models.

## Discussion

This work addresses the need of the biological research community for rigorously quantitative and generally applicable computational tools to compare the complex behaviors of individual members of groups of molecules, cells or even organisms. Presently, the vast majority of such comparisons are performed manually, or "by eye". As such, they are time-consuming, susceptible to inadvertent bias and errors and can be insensitive to subtleties. Using the regulatory effects of the microtubule associated protein tau upon the dynamic behavior of microtubules as a system of study, we have developed a novel modeling and visualization strategy allowing investigators (i) to assess the relative degree of similarity/dissimilarity among individual tau isoforms with respect to numerous parameters of interest under varying experimental conditions, and (ii) to visualize all the conditions with respect to each other. More importantly, the same computational strategy should be generally applicable to a great many other applications.

The validity of the two-dimensional embedding strategy presented in this paper is established by comparing the plot presented in Figure 3 with the growth rate data in Table 1. The relative positions of all points in Figure 3 are in complete agreement with the quantitative growth rate data determined both automatically and manually. Additionally, the semi-quantitative analysis of the histograms shown in Figure 4 of [19] are confirmed and extended by the more rigorous quantitative analysis leading to the two-dimensional embedding plot shown in Figure 5. In this case, 19 different bins of microtubule growth rates were integrated into the analysis for each of the nine experimental conditions tested. The resulting two-dimensional plot in Figure 5 presents the investigator with novel perspectives on the data set, including the existence of two clusters of histogram distributions based on growth rate as well as the distinct behavior of low ratios of 3-repeat tau:tubulin relative to all other tested reactions.

Finally, although the molecular mechanisms underlying behaviors suggested by various statistical models may not be clear, these models could suggest mechanisms that could not be drawn using the standard manual analytical methods generally utilized in biological investigations. Indeed, one of the most important and generally applicable features of our computational strategy is the ability to detect subtle relationships between different molecules or conditions that might escape manual investigation.

## Conclusion

In this manuscript, we present (i) an automated method for quantitatively characterizing microtubule dynamics as a function of time, and (ii) a novel and generally applicable computational tool for two-dimensional visualization and modeling of entities of interest for comparative studies. Comparison of our automated tracking method with manually acquired data demonstrates its accuracy. This tool greatly increases the rate at which microtubule tracking data can be acquired as well as improve upon its objectivity and accuracy. Our embedding strategy accurately recapitulates and extends previous biological observations that were collected and analyzed manually. Importantly, our methods facilitate the integration of sophisticated statistical modeling with biological investigations, which should promote novel and deeper mechanistic insights into biological phenomena as well as the development of testable hypotheses for subsequent investigation. In the future, we anticipate applying these methods to compare wild-type tau action versus various tau mutants causing neurodegeneration and dementia, seeking to identify novel mechanistic effects. Additionally, we envision using new models and embedding strategies.

## Methods

### Modeling

The different models described in this section capture different characteristics of microtubule dynamicity. Comparison of conditions across these models highlights different features of tau action.

#### Microtubule Events

Three kinds of events are used to characterize microtubule dynamics: growth, shortening and attenuation ("pause"). Each kind of event can be *simple *or *complex*. An event is simple when it is characterized between two consecutive tracked time-points. Simple events are coalesced together to form bigger complex events. Complex events, therefore, can be defined over a contiguous set of more than two time-points. Identification of simple events are easy, but identifying the start and end points of complex events require sub-sequence analysis.

The simple events are classified in the following manner. The different parameters for defining these events used in this particular study are indicated next. An event is a *simple growth *if and only if: (i) the rate of increase of microtubule length is at least 0.5 *μm*/*min*, and (ii) the increase in length is at least 0.05 *μm*. The corresponding parameters for a *simple shortening *event are: (i) the rate of decrease of length is at least 0.5 *μm*/*min*, and (ii) the decrease in length is at least 0.5 *μm*. A *simple attenuation *event must have (i) a rate of change of length outside the range for simple growth and simple shortening events, i.e., between -0.5 *μm*/*min *and +0.5 *μm*/*min*, and (ii) a total time duration of at least 4 *s*. Any event that does not fall in any of the above categories are *excluded *from the analysis. Due to errors in human tracking and image resolution issues, such events are likely to be part of the input noise, and are hence, discarded. Simple events are used for Markov Chain analysis.

The complex events have their corresponding parameter cut-offs as well. However, the more important consideration in the analysis of complex events is the identification of where it starts and where it ends. A survey of such methods from the time-series literature can be found in [37]. These methods have been successfully used to segment time-series streams into different partitions in various application domains, most notably for stock market analysis. An interesting way to combine different segmentation outputs has been proposed in [38]. However, none of these methods have employed priority rules to analyze adjacency relationships.

We now describe our bottom-up approach of identifying complex events by merging together simple events. First, all consecutive simple events of the same type are merged together to form a longer complex event of the same type. Next, each complex event is subjected to the rule set for classifying into growth, shortening, and attenuation. An event that does not pass any of the three rule sets is classified temporarily as an error. Also, the cause of its failure is noted. More specifically, any event where there is an increase in length but which cannot be classified as a growth is assigned into two kinds of failure: (i) rate, where it did not pass the growth rate threshold, and (ii) length, where it did not pass the growth length threshold. The failed shortening events are classified similarly. Note that there are no attenuation failure classes.

The priority rules are applied next. A growth rate failure event is most likely to be part of an attenuation. Thus, its neighbors are examined and if possible, it is combined with adjacent attenuation events to form a bigger attenuation event. If this fails, then attempts to incorporate with neighboring growth events, if any, are made. If, however, the growth failure event is due to the length cutoff and not the rate, then this event is most likely to be part of a growth event. The error in length may be due to human tracking and image resolution issues. Hence, attempts to combine this with neighboring growth events are first carried out. The rules for absorbing the shortening failure events are similar.

The complex event cut-offs are: (i) Growth: rate ≥ 0.5 *μm*/*min*, length ≥ 0.06 *μm*; (ii) Shortening: rate ≤ -0.5 *μm*/*min*, length ≤ -0.6 *μm*; and (iii) Attenuation: rate between -0.5 *μm*/*min *and +0.5 *μm*/*min*, time ≥ 30 *s*. The growth rates and the growth distributions are calculated using the complex events.

Figure 2 shows a comparison of the manually marked complex events and the automatically measured ones. The solid line indicates the simple events. As evident from the figure, these tracked lengths are noisy. The complex events get rid of the noise by smoothing over a range of simple events. However, while the automatic method marks three events – two growth events separated by a shortening event – a human may simply mark the entire time-history as a single growth event. Clearly, this human bias will differ from one experimenter to another, and may even vary from time to time. Note that this explains why growth rates obtained from the automatic measurements vary (become slightly higher) from those obtained through the manual method.

The parameters for the different events have been chosen empirically by biologists based on experimenting with different kinds of microtubule samples. The event definitions have been used consistently and have become the *de facto *"industry standard," as evident from [4].

#### Growth Rate

The growth rate for a particular experimental condition was calculated as the average of the growth rates of all the complex growth events of the microtubules for that condition.

In order to understand whether the differences between the automatically computed growth rate values using the above event analysis technique and the manually measured ones are statistically significant, we calculated the *p*-*values *in the following way. Two groups were formed, one with the automatically identified growth events, and the other with the manually marked growth events. We then performed a t-test [39] to determine whether the means of the growth rates of the two groups are different. The p-values thus obtained are reported in Tables 1 and 2.

#### Growth Rate Distribution Histogram

For each condition, a growth rate distribution histogram was computed in the following manner. The rates for the complex growth events were divided into 18 bins of width 0.4 *μm*/*min *each (consistent with analysis by [19]), starting from 0.5 *μm*/*min *up to 7.7 *μm*/*min*. Once again, these parameters conform to the standards set in the microtubule event analysis literature [4]. All the higher growth rate events were collected in another bin. Thus, the histograms had 19 bins in total. The bin heights were normalized such that they add up to 1, yielding a growth rate distribution. In order to generate histograms with a fixed number of bins, say *b*, the width of each bin was specified as $\frac{7.7-0.5}{b}\mu m/min$.

#### Haar Wavelets

Wavelets are mathematical functions that describe time-series data in terms of various frequency components with resolutions matched to their scales [31]. The orthonormal basis vectors, called the *mother wavelets*, that describe the various wavelet components are given by:

*ψ*_*s,l*_(*x*) = 2^-*s*/2^*ψ*(2^-*s *^*x *- *l*)

where *s *denote the scaling factor and *l *the localization in time. The Haar wavelet basis functions [30] are the simplest:

$$\psi (x)=\{\begin{array}{ll} +1 & 0\leq x<1/2\\ -1 & 1/2\leq x<1\\ 0 & \text{otherwise} \end{array}$$

Haar wavelets are also the fastest to calculate with respect to other wavelet bases. They work by progressively retaining the most important parameters of a signal. The first coefficient is the "sum" (actually, scaled average) of the entire signal and the next gives the "detail" (difference of the two halves) of the signal. The later coefficients give more and more details about each half of the signal they model.

In general, more wavelet parameters mean more detailed description; however, they also mean more data and more noise. Further, the error in embedding is directly proportional to the number of parameters of the original data. Thus, 16 coefficients of each microtubule time-series were retained. These 16 coefficients were then averaged over all the microtubules from a particular experimental condition to yield the coefficients for that condition.

#### Markov Chain

A *Markov Chain *(MC) [32] is a discrete time stochastic process that models the observations of a dynamic system (such as the growth or the shortening of a microtubule) as the states of the system. The number of states is finite and there is a state corresponding to each observation symbol. In a first-order MC, the probability of occurrence of the future state (or observation) depends only on the current state; past states are inconsequential. This property is called the *Markov property*. (In a *k*^th ^order Markov Chain, the future state depends on the current state and *k *- 1 past states.)

Formally, an MC *λ *is defined as:

*λ *= {*n*, *π*, *τ*}

where *n *is the *number of states*, *π *is the *start state probability vector *of length *n*, and *τ *is the *n *× *n transition matrix*; *π*(*u*) denotes the probability of being in state *u *in the first time-step; and, *τ *(*u, v*) denotes the probability of reaching state *v *from state *u *in a single time-step.

In the work of [19], microtubules were in a non-equilibrium phase, exhibiting very little shortening and many microtubules never shortened at all. Therefore, the microtubule events were discretized into two symbols: *G *for growth, and *N *for non-growth (shortening or attenuation). The Markov Chains were built with these two states – growth and non-growth. Since shortening events were very rare, modeling it as a separate state would have lacked statistical validity.

The transition probabilities for the MCs were estimated in the following manner. Every microtubule time-series was denoted as a string of symbols, with each symbol representing a simple event. Then, pairs of consecutive symbols (states) were read and appropriate entries in the transition matrix were incremented. When all the microtubules in an experimental condition were processed, the transition matrix was normalized such that the sum of transition probabilities from each state form a probability distribution (adds up to 1). The start state probabilities were estimated in a similar manner by reading the first symbol for every microtubule time-series; if it is growth, the entry for *G *is incremented, otherwise that for *N *is incremented. Finally, normalization was performed such that the probabilities add up to 1. Since most of the microtubules started with growth, these vectors were very close to [1, 0].

#### Lomb-Scargle Periodograms

The periodicity analysis of the microtubule data was performed by extracting Lomb-Scargle coefficients [33,34] from each time-series. Lomb-Scargle periodograms capture the different frequency components in a time-series and can handle missing values and unequal sampling intervals. Four low frequency components (corresponding to periodicities of 4, 8, 16, and 32 s) were retained for each microtubule. The Lomb-Scargle coefficients for the condition were computed as the average of the corresponding coefficients of the individual microtubules.

### Dissimilarity Functions

In order to compare a pair of models, an appropriate similarity or dissimilarity function is necessary. The dissimilarity or distance measure is used to compute the distance matrix among the conditions; this distance matrix is then embedded in a two-dimensional vector space as described later in the Visualization section.

#### Growth Rate

The dissimilarity between a pair of conditions with respect to the growth rates was measured by their *difference*. The difference can be also viewed as a *Minkowski *form of distance or *L*_*k *_norm. The *L*_*k *_norm between two vectors *p *and *q *of length *k *each is defined as:

$$L_{k}(p,q)={\left[\sum_{\forall i}{\left|p_{i}-q_{i}\right|}^{k}\right]}^{1/k}$$

#### Growth Rate Distribution Histogram

Growth rate histograms can be viewed as vectors and *L*_*k *_norms can be employed to capture the dissimilarity between a pair of histograms. These measures, however, do not capture the relationship among the different histogram bins. For example, suppose there are three bins in each histogram corresponding to low rate of growth, medium rate of growth and high rate of growth. If *A *= [1, 0, 0], *B *= [0, 1, 0] and *C *= [0, 0, 1], then *L*_*k *_norms treat these histograms as equidistant from each other, even though *A *should be more different from *C *than *B*. To capture such spatial properties of the bins, *match distance *[29,40] was employed.

To calculate the match distance between a pair of histograms p and q, a distance matrix among the bins of the histogram are specified – the distance between two bins *i *and *j *is *c*_*ij *_= |*i *- *j*|. The match distance is defined as the minimum work required to be done in order to transform the histogram *p *into the histogram *q *by moving values or "flows" from the bins of *p *to those in *q *and vice versa. Having a flow *f*_*ij *_from bin *i *of *p *to bin *j *of *q *or vice versa is considered as *c*_*ij*·_*f*_*ij *_amount of work. Finding the match distance then reduces to finding the flows *f*_*ij *_such that the total work done is minimum. The minimum work done is the match distance:

$$MD(p,q)=\underset{f}{\min}\left\{\sum_{i,j}c_{ij}f_{ij}\right\}$$

For the example histograms *A*, *B*, and *C *mentioned above, the match distances are *MD*(*A, B*) = 1, *MD*(*B, C*) = 1, and *MD*(*A, C*) = 2. Clearly, this captures the relatively larger dissimilarity of *A *from *C *as compared to that from *B*.

For one-dimensional histograms where the sum of the bin values add up to the same number (here, 1), match distance can be calculated more easily as the *L*_1 _distance between the cumulative bin values of the two histograms:

*MD*(*p, q*) = *L*_1_(*P, Q*)

where $P_{i}=\sum_{j=1}^{i}p_{i}$ and $Q_{i}=\sum_{j=1}^{i}q_{i}$ are the cumulative histogram bin values.

#### Haar Wavelets

Since the relative importance of the wavelet parameters differ, a simple distance function such as *L*_1 _would be inappropriate. Coefficients that summarize the entire time-series, such as the "sum" value and the overall "detail" value is more important, and therefore, should get higher weights than the coefficients describing parts of the time-series.

Thus, in order to determine the dissimilarity between two conditions with respect to their Haar wavelet coefficients, we used the *weighted L*_1 _norm or the *weighted Manhattan *distance. The levels of the wavelet tree were weighted such that the overall sum and the overall detail coefficient were the most significant values, the next level detail coefficients getting an exponentially lower weight and so on. The weight vector, of length 16, was [8, 8, 4, 4, 2, 2, 2 2, 1, 1, 1, 1, 1, 1, 1, 1]. For two vectors *p *and *q*, and a weight vector *w*, all of length *k*, the weighted *L*_1 _distance between *p *and *q *is measured as:

$$L_{1}(p,q)=\sum_{i=1}^{k}w_{i}\left|p_{i}-q_{i}\right|$$

The *L*_2 _norm or the Euclidean distance was applied to measure the distances between a pair of conditions for both the Markov Chain parameters and the Lomb-Scargle coefficients.

### Visualization

The distances among the experimental conditions, calculated by using the above methods, were visualized by plotting the conditions onto a two-dimensional vector space. This allows for easy comparison of the conditions and immediate comprehension of the structure of the data. The aim of the embedding method is to assign coordinates such that the Euclidean distance between any pair of conditions in the embedded space is as close as possible to the dissimilarity calculated between their models. The method can embed a given set of points into any dimensional space; here, we have chosen two for easy visualization purposes.

Formally, suppose there are two models, *α *and *β*, and the dissimilarity between them is *d*(*α*, *β*) according to some dissimilarity function *d*. If the embeddings of these two models in the two-dimensional vector space (*x*, *y *space) is given by *e*(*α*) = (*x*_*α*_, *y*_*α*_) and *e*(*β*) = (*x*_*β*_, *y*_*β*_), then the aim of the embedding function is to choose the coordinates *e*(*α*) and *e*(*β*) such that the relative difference between *L*_2_(*e*(*α*), *e*(*β*)) and *d*(*α*, *β*) is minimum. When there are n such models, the embedding function should be chosen such that the relative cumulative difference for all the *n*(*n *- 1)/2 pairs is minimized.

Principal component analysis (PCA) [28] can also be used to project data onto a two-dimensional space. PCA chooses the axes along which the original data shows the highest variance. It does not take into account the distances among the points. More importantly, PCA cannot work with any general distance matrix and is used mostly as a dimensionality reduction technique.

We used the Sammon projection method [27] as the embedding procedure. This method has been successfully used to embed proteins on a two-dimensional space for clustering purposes [41]. The method starts with a random point (random *x*, *y *coordinates) for each model. In each iteration, the points are updated according to a *steepest descent *algorithm such that the following *error *function *E*(*x*, *y*), which measures the relative differences between the original distances and the embedded distances, is decreased.

$$E(x,y)=\frac{1}{c}\sum_{\forall \alpha ,\beta ,\alpha \neq \beta }\left[\frac{{\left(d(\alpha ,\beta )-L_{2}(e(\alpha ),e(\beta ))\right)}^{2}}{d(\alpha ,\beta )}\right]$$

where

$$c=\sum_{\forall \alpha ,\beta }d(\alpha ,\beta )$$

In each iteration, a correction step is added to every dimension of every point. The direction of the correction is towards the gradient of the error. The coordinates of the point *α *in iteration *i *+ 1 are updated as follows:

$$\begin{array}{c} x_{\alpha }^{\left(i+1\right)}=x_{\alpha }^{\left(i\right)}-f\times \frac{\partial E^{\left(i\right)}/\partial x}{\left|\partial^{2}E^{\left(i\right)}/\partial x^{2}\right|}\\ y_{\alpha }^{\left(i+1\right)}=y_{\alpha }^{\left(i\right)}-f\times \frac{\partial E^{\left(i\right)}/\partial y}{\left|\partial^{2}E^{\left(i\right)}/\partial y^{2}\right|} \end{array}$$

where *E*^(*i*) ^is the error after iteration *i *and *f *is a factor to control the step sizes. We used *f *= 0.2. The method stops after a certain number of steps or when there is no significant improvement in the error. We stopped the iterations either when the change in error went below 0.01% or up to a maximum of 1000 steps. The number of steps was set as an additional check in order to come out of any local error problems, e.g., oscillating error values. In practice, after 250–300 iterations, the error stopped changing, and the algorithms stopped. In addition, in order to counter the problem of bad initialization, the algorithms were run 5 times for each embedding and the one with the lowest error was picked. The final coordinates or the directions of the axes do *not *have any significance; *only *the Euclidean distances among the embedded points matter.

For any dimensionality reduction or embedding technique, an important measure of quality is *distortion*. Distortion measures the largest amount of discrepancy from an original distance value to the corresponding embedded distance. It is measured as

$$distortion=\frac{\max\left\{\frac{original\;dist}{embedded\;dist}\right\}}{\min\left\{\frac{original\;dist}{embedded\;dist}\right\}}$$

where *original dist *refers to an original dissimilarity measure between two models and *embedded dist *refers to the Euclidean distance between the corresponding embedded points. For ideal embeddings, where all the original distances have been maintained exactly, the distortion is 1. For others, the distortion is greater than 1. In general, lower the distortion value, better the embedding. The individual distortions are measured by the ratio of *embedded dist *to *original dist*.

For models with only a single parameter, any dissimilarity between a pair of them is equal to the difference between their single parameters. Such distance matrices can be always embedded into two dimensions with distortion equal to 1. The models have to be simply embedded as points on a straight line with the order and the distances maintained, e.g., as (*parameter*, 0) or (*parameter*/$\sqrt{2}$, *parameter*/$\sqrt{2}$) points. In our implementation, we have not forced this explicitly; the method itself converges to a straight line plot. For models with two parameters, if the dissimilarity function is Euclidean, then, it is again possible to devise an embedding with distortion 1. The original parameter values will form the coordinates in the embedded space. For higher number of parameters or with other dissimilarity functions, in general, it is not possible to design embeddings with distortion 1. The distortions of each of the graphs are mentioned in the captions. Additional file 3 reports the individual distortions for each of the distances for all the embeddings.

## Authors' contributions

AB built the models, computed the pairwise distances, and embedded them on two dimensions for comparisons. AL, SL and MG collected the data and manually tracked the microtubules. AKS advised on the choice of the models and the distance functions. SCF and LW provided the biological interpretations. AB and SCF wrote the manuscript with inputs from the other authors. All the authors read and approved the final manuscript.

## Supplementary Material

Additional file 1 This file shows the effect of number of bins on the growth rate distribution histograms. Click here for file

Additional file 2 This file shows the two-dimensional embedding plots of the Haar wavelet coefficients of microtubules with varying tau:tubulin molar ratios for both the samples. Click here for file

Additional file 3 This file shows the individual distortions of all the pairwise distances between the conditions for all the different models described. Click here for file

## References

[B1] Alberts B, Johnson A, Lewis J, Raff M, Roberts K, Walter P (2002). Molecular Biology of the Cell. Garland Science.

[B2] Mitchison T, Kirschner M (1984). Dynamic Instability of Microtubule Growth. Nature.

[B3] Jordan MA, Wilson L (2004). Microtubules as a Target for Anticancer Drugs. Nature Reviews Cancer.

[B4] Feinstein SC, Wilson L (2005). Inability of Tau to Properly Regulate Neuronal Microtubule Dynamics: A Loss-of-function Mechanism by which Tau might Mediate Neuronal Cell Death. Biochimica et Biophysica Acta.

[B5] Yvon AMC, Wadsworth P, Jordan MA (1999). Taxol Suppresses Dynamics of Individual Microtubules in Living Human Tumor Cells. Molecular Biology of Cell.

[B6] Caceres A, Kosik KS (1990). Inhibition of Neurite Polarity by Tau Antisense Oligonucleotides in Primary Cerebellar Neurons. Nature.

[B7] Esmaeli-Azad B, McCarty JH, Feinstein SC (1994). Sense and Antisense Transfection Analysis of Tau Function: Tau Influences Net Microtubule Assembly, Neurite Outgrowth and Neuritic Stability. J Cell Science.

[B8] Dawson HN, Ferreira A, Eyster MV, Ghoshal N, Binder LI, Vitek MP (2001). Inhibition of Neuronal Maturation in Primary Hippocampal Neurons from Tau Deficient Mice. J Cell Science.

[B9] Cleveland DW, Hwo SY, Kirschner MW (1977). Purification of Tau, a Microtubule-Associated Protein that Induces Assembly of Microtubules from Purified Tubulin. J Molecular Biology.

[B10] Connolly JA, Kalnins VI, Cleveland DW, Kirschner MW (1977). Immunoflourescent Staining of Cytoplasmic and Spindle Microtubules in Mouse Fibroblasts with Antibody to t Protein. Proc Natl Acad Sci USA.

[B11] Drechsel DN, Hyman AA, Cobb MH, Kirschner MW (1992). Modulation of the Dynamic Instability of Tubulin Assembly by the Microtubule-Associated Protein Tau. Molecular Biology of the Cell.

[B12] Trinczek B, Biernat J, Baumann K, Mandelkow EM, Mandelkow E (1995). Domains of Tau Protein, Differential Phosphorylation, and Dynamic Instability of Microtubules. Molecular Biology of Cell.

[B13] Panda D, Samuel JC, Massie M, Feinstein SC, Wilson L (2003). Differential Regulation of Microtubule Dynamics by 3-Repeat and 4-Repeat Tau: Implications for Normal Neuronal Development and the Onset of Neurodegenerative Disease. Proc of the National Academy of Sciences USA.

[B14] Kosik KS, Orecchio LD, Bakalis S, Neve RL (1989). Developmentally Regulated Expression of Specific Tau Sequences. Neuron.

[B15] Stoothoff WH, Johnson GV (2005). Tau Phosphorylation: Physiological and Pathological Consequences. Biochimica et Biophysica Acta.

[B16] Goode BL, Feinstein SC (1994). Identification of a Novel Microtubule Binding and Assembly Domain in the Developmentally Regulated Inter-repeat Region of Tau. J Cell Biology.

[B17] Goode BL, Denis PE, Panda D, Radeke MJ, Miller HP, Wilson L, Feinstein SC (1997). Functional Interactions between the Proline-rich and Repeat Regions of Tau Enhance Microtubule Binding and Assembly. Mol Biol Cell.

[B18] Goode BL, Chau M, Denis PE, Feinstein SC (2000). Structural and Functional Differences between 3-repeat and 4-repeat Tau Isoforms: Implications for Normal Tau Function and the Onset of Neurodegenetative Disease. J Biological Chemistry.

[B19] Levy SF, LeBoeuf AC, Massie MR, Jordan MA, Wilson L, Feinstein SC (2005). Three-and Four-Repeat Tau Regulate the Dynamic Instability of Two Distinct Microtubule Subpopulations in Qualitatively Different Manners: Implications for Neurodegeneration. J Biological Chemistry.

[B20] Kosik KS, Joachim CL, Selkoe DJ (1986). Microtubule-associated Protein Tau(t) is a Major Antigenic Component of Paired Helical Filaments in Alzheimer Disease. Proc Natl Acad Sci USA.

[B21] Clark LN, Poorkaj P, Wszolek Z, Geschwind DH, Nasreddine ZS, Miller B, Li D, Payami H, Awert F, Markopoulou K, Andreadis A, D'Souza I, Lee VM, Reed L, Trojanowski JQ, Zhukareva V, Bird T, Schellenberg G, Wilhelmsen KC (1998). Pathogenic Implications of Mutations in the Tau Gene in Pallido-ponto-nigral Degeneration and Related Neurodegenerative Disorders Linked to Chromosome 17. Proc Natl Acad Sci.

[B22] Hutton M, Lendon CL, Rizzu P, Baker M, Froelich S, Houlden H, Pickering-Brown S, Chakraverty S, Isaacs A, Grover A, Hackett J, Adamson J, Lincoln S, Dickson D, Davies P, Petersen RC, Stevens M, de Graaff E, Wauters E, van Baren J, Hillebrand M, Joosse M, Kwon JM, Nowotny P, Che LK, Norton J, Morris JC, Reed LA, Trojanowski J, Basun H, Lannfelt L, Neystat M, Fahn S, Dark F, Tannenberg T, Dodd PR, Hayward N, Kwok JB, Schofield PR, Andreadis A, Snowden J, Craufurd D, Neary D, Owen F, Oostra BA, Hardy J, Goate A, van Swieten J, Mann D, Lynch T, Heutink P (1998). Association of Missense and 5'-splice-site Mutations in Tau with the Inherited Dementia FTDP-17. Nature.

[B23] Spillantini MG, Murrell JR, Goedert M, Farlow MR, Klug A, Ghetti B (1998). Mutation in the Tau Gene in Familial Multiple System Tauopathy with Presenile Dementia. Proc Natl Acad Sci.

[B24] Hong M, Zhukareva V, Vogelsberg-Ragaglia V, Wszolek Z, Reed L, Miller BI, Geschwind DH, Bird TD, McKeel D, Goate A, Morris JC, Wilhelmsen KC, Schellenberg GD, Trojanowski JQ, Lee VM (1998). Mutation-Specific Functional Impairments in Distinct Tau Isoforms of Hereditary FTDP-17. Science.

[B25] Bunker JM, Wilson L, Jordan MA, Feinstein SC (2004). Modulation of Microtubule Dynamics by Tau in Living Cells: Implications for Development and Neurodegeneration. Mol Biol Cell.

[B26] Drubin DG, Feinstein SC, Shooter EM, Kirschner MW (1985). Nerve Growth Factor-induced Neurite Outgrowth in PC12 Cells Involves the Coordinate Induction of Microtubule Assembly and Assembly-promoting Factors. J Cell Biol.

[B27] Sammon JWJ (1969). A Nonlinear Mapping for Data Structure Analysis. IEEE Trans on Computers.

[B28] Jolliffe IT (2002). Principal Component Analysis.

[B29] Werman M, Peleg S, Rosenfeld A (1985). A Distance Metric for Multi-Dimensional Histograms. Computer, Vision, Graphics, and Image Processing.

[B30] Zimmermann G Fundamental Papers in Wavelet Theory, Princeton University Press 2006 chap On the Theory of Orthogonal Function Systems.

[B31] Daubechies I (1992). Ten Lectures on Wavelets. Society for Industrial and Applied Mathematics(SIAM).

[B32] Bolch G, Greiner S, de Meer H, Trivedi KS (1998). Queueing Networks and Markov Chains.

[B33] Lomb NR (1976). Least-squares Frequency Analysis of Unequally Spaced Data. Astrophysics and Space Science.

[B34] Scargle JD (1982). Studies in Astronomical Time Series Analysis. II – Statistical Aspects of Spectral Analysis of Unevenly Spaced Data. Astrophysical Journal, Part 1.

[B35] Oppenheim AV, Schafer RW (1975). Digital Signal Processing.

[B36] Box G, Jenkins GM, Reinsel GC (1994). Time Series Analysis: Forecasting and Control.

[B37] Keogh EJ, Chu S, Hart D, Pazanni MJ (2001). An Online Algorithm for Segmenting Time Series. In Proc IEEE Int Conf on Data Mining (ICDM).

[B38] Mielikäinen T, Terzi E, Tsaparas P (2006). Aggregating Time Partitions. In Proc ACM SIGKDD Int Conf on Knowledge Discovery and Data Mining (KDD).

[B39] Press WH, Flannery BP, Teukolsky SA, Vetterling WT (1992). Numerical Recipes in C.

[B40] Peleg S, Werman M, Rom H (1989). A Unified Approach to the Change of Resolution: Space and Gray-Level. IEEE Trans on Pattern Analysis and Machine Intelligence.

[B41] Apostol I, Szpankowski W (1999). Indexing and Mapping of Proteins Using a Modified Nonlinear Sammon Projection. J Computational Chemistry.

