# Supplementary material for: A general modeling and visualization tool for comparing different members of a group: application to studying tau-mediated regulation of microtubule dynamics
Source: BMC Bioinformatics. 2008 Aug 12;9:339. doi: 10.1186/1471-2105-9-339 (PMC2533028; doi:10.1186/1471-2105-9-339)

Effect of number of bins on growth rate histogram embedding plots

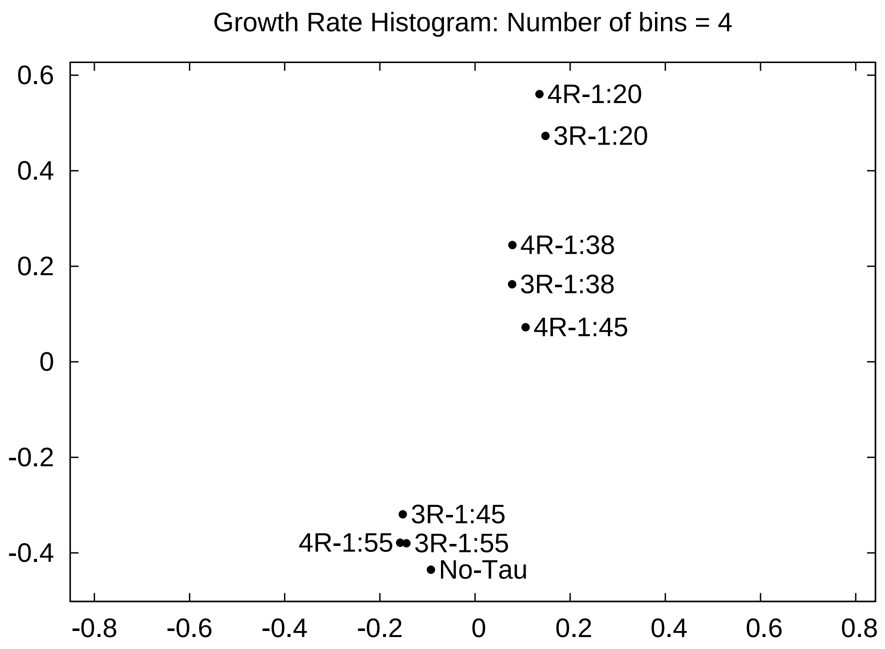

Growth Rate Histogram: Number of bins = 9

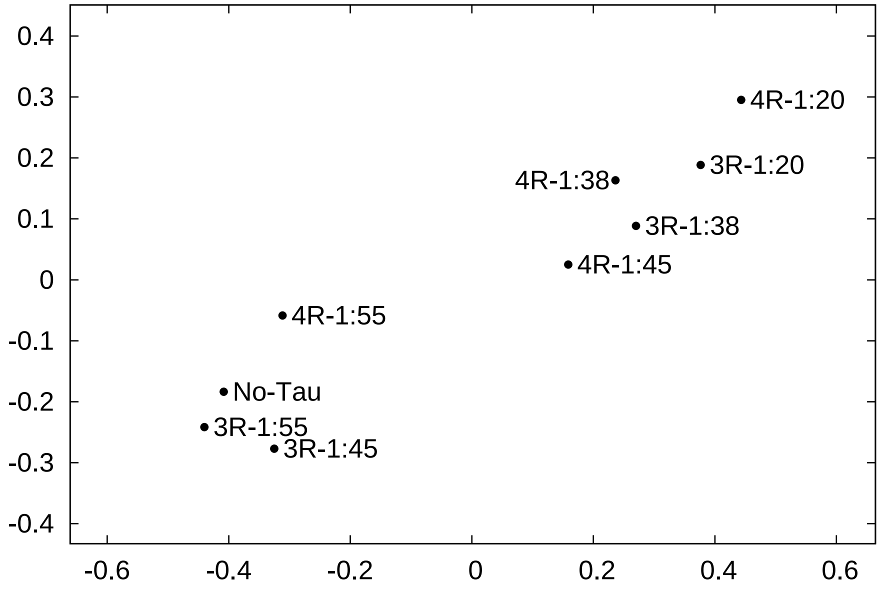

Growth Rate Histogram: Number of bins = 14

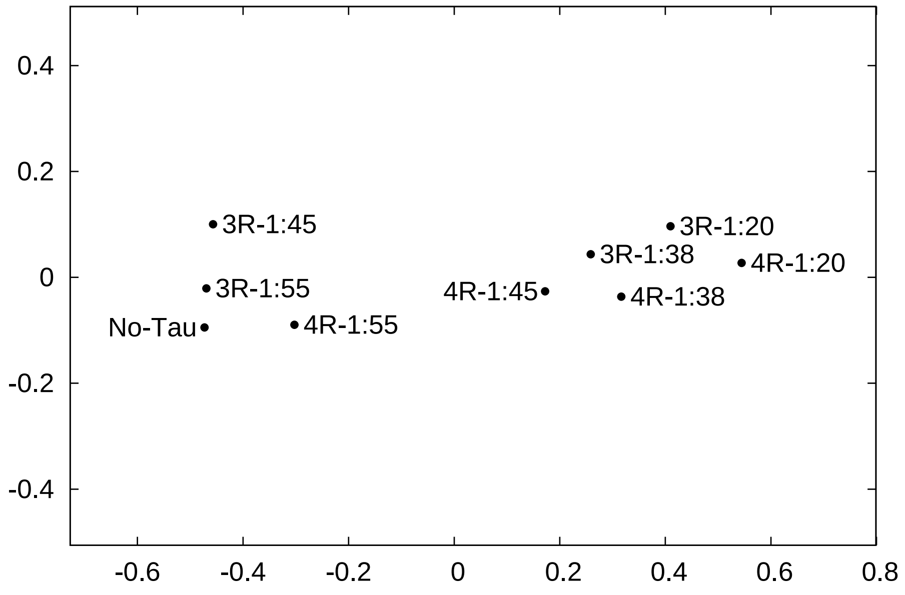

Growth Rate Histogram: Number of bins = 19

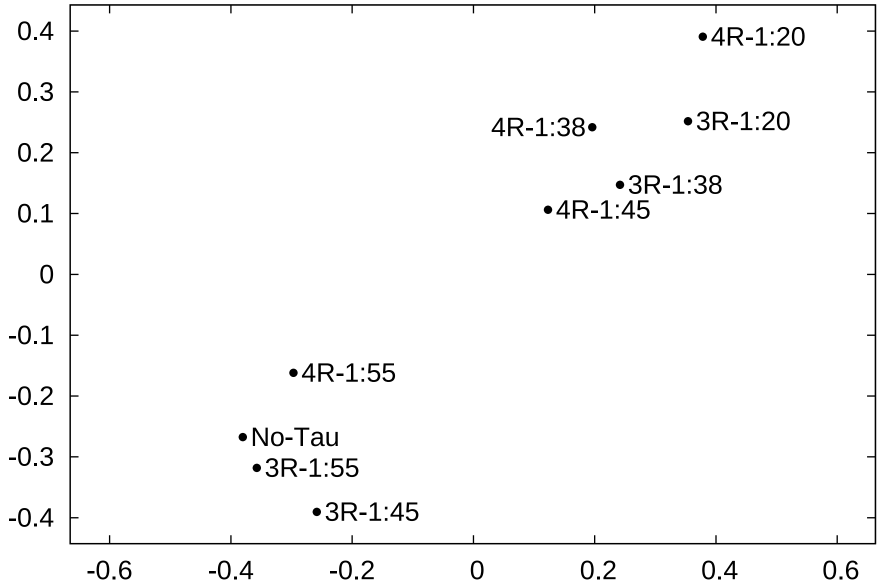

Growth Rate Histogram: Number of bins = 24

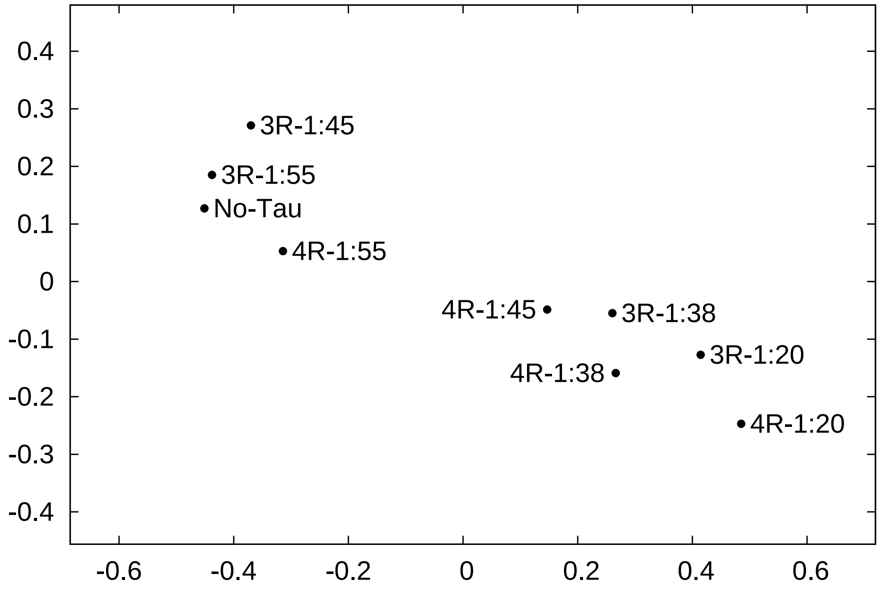

Growth Rate Histogram: Number of bins = 29

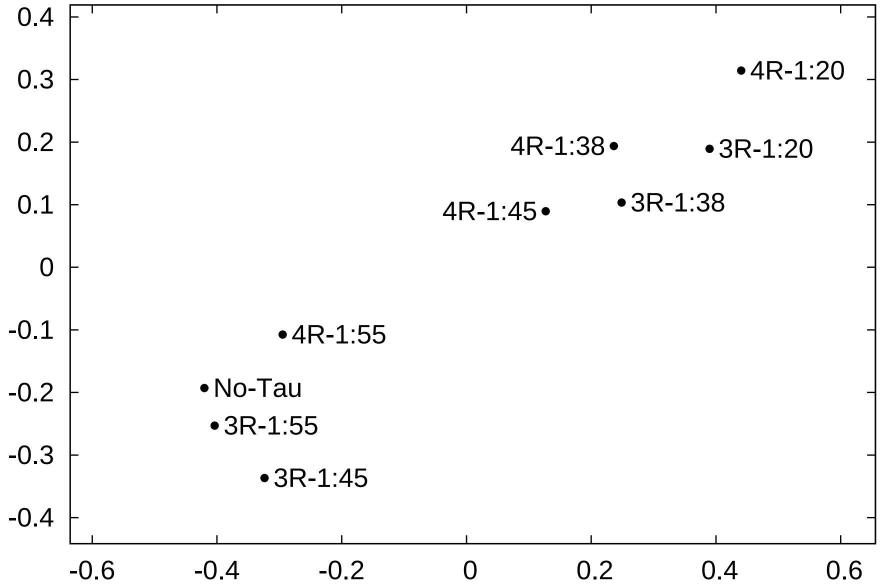

Supplement: Additional file 1 — This file shows the effect of number of bins on the growth rate distribution histograms. [file 1471-2105-9-339-S1.pdf]
