# Supplementary material for: A general modeling and visualization tool for comparing different members of a group: application to studying tau-mediated regulation of microtubule dynamics
Source: BMC Bioinformatics. 2008 Aug 12;9:339. doi: 10.1186/1471-2105-9-339 (PMC2533028; doi:10.1186/1471-2105-9-339)
Supplement: Additional file 2 — This file shows the two-dimensional embedding plots of the Haar wavelet coefficients of microtubules with varying tau:tubulin molar ratios for both the samples. [file 1471-2105-9-339-S2.pdf]

Two-dimensional embeddings of Haar wavelet coefficients

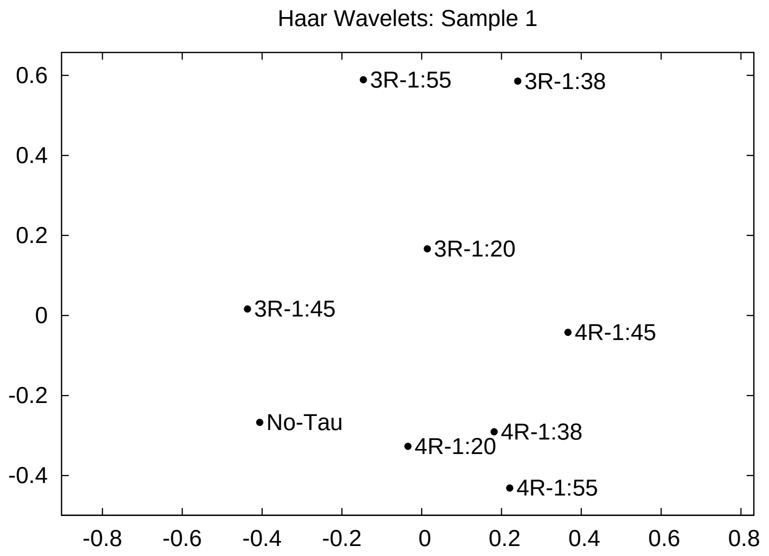

Sample 1 (Distortion = 1.79).

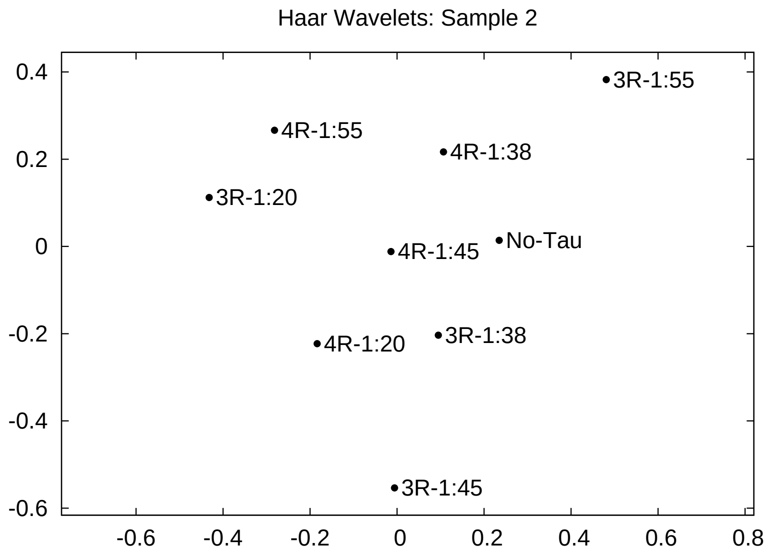

Sample 2 (Distortion = 1.94).
