# Supplementary material for: A general modeling and visualization tool for comparing different members of a group: application to studying tau-mediated regulation of microtubule dynamics
Source: BMC Bioinformatics. 2008 Aug 12;9:339. doi: 10.1186/1471-2105-9-339 (PMC2533028; doi:10.1186/1471-2105-9-339)
Supplement: Additional file 3 — This file shows the individual distortions of all the pairwise distances between the conditions for all the different models described. [file 1471-2105-9-339-S3.pdf]

Individual distortion values for the different embeddings

	3R-1:20	3R-1:38	3R-1:45	3R-1:55	4R-1:20	4R-1:38	4R-1:45	4R-1:55	No-Tau
3R-1:20	1.00	1.00	1.00	1.00	1.00	1.00	1.00	1.00	1.00
3R-1:38	1.00	1.00	1.00	1.00	1.00	1.00	1.00	1.00	1.00
3R-1:45	1.00	1.00	1.00	1.00	1.00	1.00	1.00	1.00	1.00
3R-1:55	1.00	1.00	1.00	1.00	1.00	1.00	1.00	1.00	1.00
4R-1:20	1.00	1.00	1.00	1.00	1.00	1.00	1.00	1.00	1.00
4R-1:38	1.00	1.00	1.00	1.00	1.00	1.00	1.00	1.00	1.00
4R-1:45	1.00	1.00	1.00	1.00	1.00	1.00	1.00	1.00	1.00
4R-1:55	1.00	1.00	1.00	1.00	1.00	1.00	1.00	1.00	1.00
No-Tau	1.00	1.00	1.00	1.00	1.00	1.00	1.00	1.00	1.00

Table 1: Distortions for embedding of growth rates. Total distortion = 1.00.

	3R-1:20	3R-1:38	3R-1:45	3R-1:55	4R-1:20	4R-1:38	4R-1:45	4R-1:55	No-Tau
3R-1:20	1.00	1.23	0.71	0.97	0.76	0.98	0.78	1.17	1.00
3R-1:38	1.23	1.00	1.17	1.05	1.08	0.67	0.71	0.74	0.89
3R-1:45	0.71	1.17	1.00	0.71	0.98	1.08	0.88	1.09	0.97
3R-1:55	0.97	1.05	0.71	1.00	1.19	1.16	1.00	1.00	0.73
4R-1:20	0.76	1.08	0.98	1.19	1.00	0.77	0.85	1.12	1.13
4R-1:38	0.98	0.67	1.08	1.16	0.77	1.00	0.92	0.99	1.05
4R-1:45	0.78	0.71	0.88	1.00	0.85	0.92	1.00	0.96	0.88
4R-1:55	1.17	0.74	1.09	1.00	1.12	0.99	0.96	1.00	0.78
No-Tau	1.00	0.89	0.97	0.73	1.13	1.05	0.88	0.78	1.00

Table 2: Distortions for embedding of growth rate distribution histograms. Total distortion = 1.84.

	3R-1:20	3R-1:38	3R-1:45	3R-1:55	4R-1:20	4R-1:38	4R-1:45	4R-1:55	No-Tau
3R-1:20	1.00	0.91	1.00	1.01	1.00	0.98	0.92	1.02	1.00
3R-1:38	0.91	1.00	1.00	0.99	1.02	1.03	1.05	0.94	1.01
3R-1:45	1.00	1.00	1.00	1.00	1.00	1.01	1.00	0.98	1.00
3R-1:55	1.01	0.99	1.00	1.00	1.00	1.02	1.01	0.97	1.00
4R-1:20	1.00	1.02	1.00	1.00	1.00	1.00	0.99	0.98	1.00
4R-1:38	0.98	1.03	1.01	1.02	1.00	1.00	0.99	0.96	1.00
4R-1:45	0.92	1.05	1.00	1.01	0.99	0.99	1.00	1.01	0.99
4R-1:55	1.02	0.94	0.98	0.97	0.98	0.96	1.01	1.00	1.01
No-Tau	1.00	1.01	1.00	1.00	1.00	1.00	0.99	1.01	1.00

Table 3: Distortions for embedding of Markov Chain models. Total distortion = 1.15.

	3R-1:20	3R-1:38	3R-1:45	3R-1:55	4R-1:20	4R-1:38	4R-1:45	4R-1:55	No-Tau
3R-1:20	1.00	0.93	0.80	0.75	0.80	1.07	0.93	1.04	0.91
3R-1:38	0.93	1.00	1.03	0.94	0.90	1.07	0.90	1.01	1.18
3R-1:45	0.80	1.03	1.00	0.96	1.02	1.06	1.06	1.11	0.79
3R-1:55	0.75	0.94	0.96	1.00	0.93	1.08	1.01	1.10	1.17
4R-1:20	0.80	0.90	1.02	0.93	1.00	0.66	0.80	0.91	0.82
4R-1:38	1.07	1.07	1.06	1.08	0.66	1.00	1.07	0.80	0.76
4R-1:45	0.93	0.90	1.06	1.01	0.80	1.07	1.00	0.88	1.15
4R-1:55	1.04	1.01	1.11	1.10	0.91	0.80	0.88	1.00	1.11
No-Tau	0.91	1.18	0.79	1.17	0.82	0.76	1.15	1.11	1.00

Table 4: Distortions for embedding of Haar wavelets. Total distortion = 1.79.

	3R-1:20	3R-1:38	3R-1:45	3R-1:55	4R-1:20	4R-1:38	4R-1:45	4R-1:55	No-Tau
3R-1:20	1.00	0.87	1.06	0.71	0.79	1.03	1.32	0.85	1.04
3R-1:38	0.87	1.00	1.02	0.91	1.12	1.01	1.14	0.85	1.06
3R-1:45	1.06	1.02	1.00	0.84	0.96	0.92	0.81	0.87	0.96
3R-1:55	0.71	0.91	0.84	1.00	0.80	0.91	0.82	0.90	0.85
4R-1:20	0.79	1.12	0.96	0.80	1.00	1.16	1.08	1.13	0.92
4R-1:38	1.03	1.01	0.92	0.91	1.16	1.00	0.97	0.89	1.08
4R-1:45	1.32	1.14	0.81	0.82	1.08	0.97	1.00	0.92	0.89
4R-1:55	0.85	0.85	0.87	0.90	1.13	0.89	0.92	1.00	1.10
No-Tau	1.04	1.06	0.96	0.85	0.92	1.08	0.89	1.10	1.00

Table 5: Distortions for embedding of Lomb-Scargle periodograms. Total distortion = 1.85.
